# Underlying Mechanisms of Chromatographic H/D, H/F, *cis/trans* and Isomerism Effects in GC-MS

**DOI:** 10.3390/metabo15010043

**Published:** 2025-01-11

**Authors:** Dimitrios Tsikas

**Affiliations:** Institute of Toxicology, Core Unit Proteomics, Hannover Medical School, 30623 Hannover, Germany; tsikas.dimitros@mh-hannover.de

**Keywords:** *cis/trans*-effect, gas chromatography, H/D-effect, H/F-effect, mass spectrometry, mechanism, partition chromatography, retention, stationary phase, Van der Waals forces

## Abstract

Charge-free gaseous molecules labeled with deuterium ^2^H (D) atoms elute earlier than their protium-analogs ^1^H (H) from most stationary GC phases. This effect is known as the chromatographic H/D isotope effect (*hd*IE_C_) and can be calculated by dividing the retention times (*t*_R_) of the protiated (*t*_R(H)_ ) to those of the deuterated (*t*_R(D)_) analytes: *hd*IE_C_ = *t*_R(H)_/*t*_R(D)_. Analytes labeled with ^13^C, ^15^N or ^18^O have almost identical retention times and lack a chromatographic isotope effect. Derivatives of *cis-* and *trans*-analytes such as *cis-* and *trans*-fatty acids also differ in their retention times. Analytes that contain *trans*-C=C-double bonds elute earlier in gas chromatography-mass spectrometry (GC-MS) than their *cis*-C=C-double bonds containing congeners. The chromatographic *cis/trans*-effect (*ct*E_C_) can be calculated by dividing the retention times of the *cis*- by those of the *trans*-analytes: *ct*E_C_ = *t*_R(c)/_*t*_R(t)_. In the present work, the *hd*IE_C_ and *ct*E_C_ values of endogenous and exogenous substances were calculated from previously reported GC-MS analyses and found to range each between 1.0009 and 1.0400. The examination suggests that the H/D-isotope effects and the *cis/trans*-effects observed in GC-MS are based on differences in the inter-molecular interaction strengths of the analyte derivatives with the stationary phase of GC columns. The deuterium atoms, being larger than the H atoms of the analytes, attenuate the interaction of the skeleton of the molecules with the GC stationary phase. The angulation of *trans*-analytes decreases the interaction of the skeleton of the molecules with the GC stationary phase, as only parts of the molecules are close enough to the GC stationary phase to interact. Other chromatographic effects caused by hydrogen (H) and fluorine (F) atoms and by stereo-isomerism are considered to be based on a similar mechanism due to the different orientation of the side chains.

## 1. Introduction

**Figure 1:**
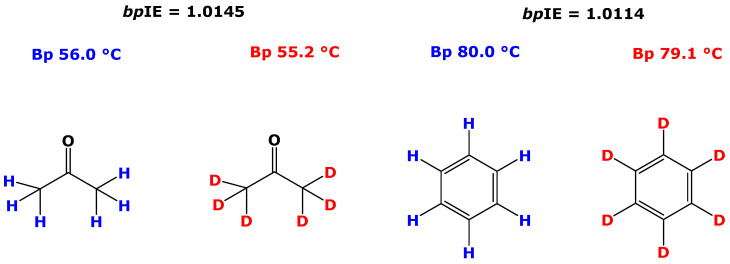
Chemical structures and boiling points (Bps) of protiated acetone (CH_3_COCH_3_), deuterated acetone (CD_3_COCD_3_), protiated benzene (C_6_H_6_) and deuterated benzene (C_6_D_6_) and their boiling point isotope effect (*bp*IE) values.

The boiling points (Bps) of chemical compounds are constant under fixed environmental conditions, notably temperature and atmospheric pressure. The Bps of many protiated and deuterated solvents such as acetone and benzene are known. The Bp of protiated acetone (CH_3_COCH_3_) is 56.0 °C and that of deuterated acetone (CD_3_COCD_3_) is 55.2 °C. The Bp of protiated benzene (C_6_H_6_) is 80.0 °C and that of deuterated benzene (C_6_D_6_) is 79.1 °C. When we define the *boiling point isotope effect*, *bp*IE, as the ratio of the Bp of protiated to deuterated solvents (Formula (1)), the *bp*IE values would be 1.0145 for acetone and 1.0114 for benzene due to the lower Bp of the deuterated solvents (Figure 1).

*bp*IE = Bp_(H)_/Bp_(D)_ (1)

The Bps of thousands of natural and synthetic analytes are unknown. Their Bp values are not only difficult to determine, but their determination would also be extremely expensive. Consequently, the boiling points, and most likely many other physicochemical parameters of analytes, seem not to be useful physicochemical measures to quantify isotope effects of analytes. On the other hand, data available for many other solvents, notably for purely inorganic water (Bp, 99.98 °C for H_2_O, Bp, 101.4 °C for D_2_O), suggest that the utility of the Bp would be of limited value.

**Figure 2:**
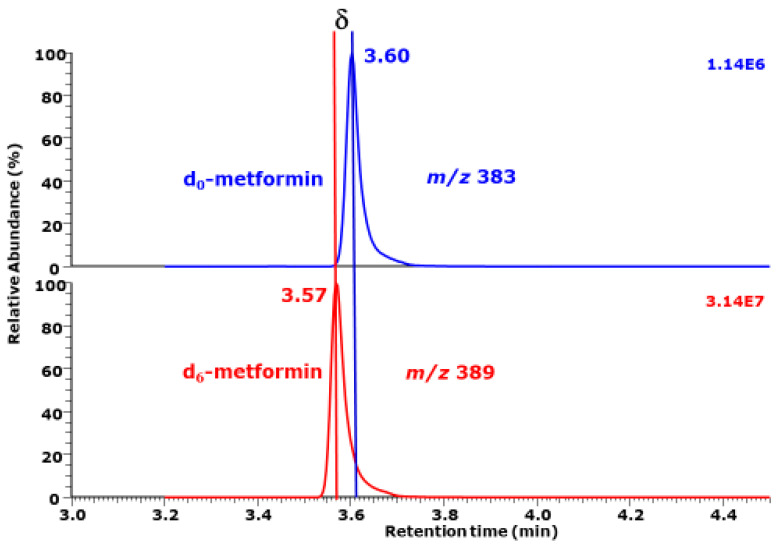
Partial GC-NICI-MS chromatogram from the quantitative analysis of the anti-diabetic drug metformin in a human serum sample as pentafluoropropionyl (PFP) derivative on an ISQ apparatus directly interfaced with a Trace 1310 series gas chromatograph. Selected-ion monitoring (SIM) of *m/z* 383 for unlabeled metformin (d_0_-metformin) and *m/z* 389 for deuterated metformin (d_6_-metformin) was performed. **δ** is the difference in the retention times of d_0_-metformin (*t*_R(H)_, 3.60 min) and d_6_-metformin (*t*_R(D)_, 3.57 min). The *hd*IE_C_ is calculated to be 1.0084 (Formula (2)). Adopted from Reference [1].

In gas chromatography-mass spectrometry (GC-MS) and liquid chromatography-mass spectrometry (LC-MS) including their tandem variants, analytes and their stable isotope labeled analogs, i.e., the isotopologs, which are used as internal standards (ISs), are separated mass spectrometrically due to their different mass-to-charge ratio (*m/z*) values. Isotopologs may also differ in their retention times, with the deuterated (D) analytes having, as a rule, shorter retention times, i.e., *t*_R(D)_, than their protiated (H), i.e., *t*_R(H)_, analogs (Figure 2) [1]. In order to discriminate this effect from other kinds of isotope effects, such as the kinetic isotope effect, this phenomenon is named in the present work the chromatographic isotope effect, IE_C_, i.e., *hd*IE_C_ for the chromatographic isotope effect due to H/D. Its extent can be determined by dividing the experimentally observed *t*_R(H)_ values by the *t*_R(D)_ values (see Formula (2)), analogous to the *bp*IE values mentioned above. The difference in the *t*_R(H)_ and *t*_R(D)_ values, i.e., δ_(H/D)_ (Figure 2), may also be useful for the determination of the extent of the chromatographic isotope effect (see Formula (3)).

*hd*IE_C_ = *t*_R(H)_/*t*_R(D)_ (2)

δ_(H/D)_ = *t*_R(H)_ − *t*_R(D)_ (3)

Based on the theory of the partition chromatography [2], the smaller retention times of ^2^H-isotopologs compared to their ^1^H-isotopologs would result from a weaker interaction strength of the ^2^H-isotopologs with the stationary phase. The closer the *hd*IE_C_ value to the unity (1.0000) is, and the smaller the δ_(H/D)_ is, the weaker is the *hd*IE_C_. 

The underlying mechanisms of chromatographic isotope effects have been little explored. Many factors are likely to contribute to *hd*IE_C_, notably including differences in the physical and chemical properties of the isotopologs (e.g., C-H/C-D bond energies, boiling points), as described above for acetone and benzene. The lower Bp values of CD_3_COCD_3_ and C_6_D_6_ compared to those of CH_3_COCH_3_ and C_6_H_6_ could be, in analogy, due to somewhat weaker inter-molecular interactions of deuterated analytes compared to their protiated congeners.

In GC-MS and LC-MS, isobaric analytes, i.e., analytes with identical *m/z* values but different stereochemistry, can be possibly separated by chromatography but not mass spectrometry. This is, for instance, the case of *cis*- and *trans*-fatty acids [3]. Empirically, *trans*-fatty acids have shorter retention times than their congeners *cis*-fatty acids in GC, thus resembling the *hd*IE_C_ effect. In analogy, the chromatographic *cis*/*trans*-effect is named in the present work the chromatographic *cis/trans*-effect*, ct*E_C_, and can be calculated by Formula (4). In this Formula, *t*_R(*cis*)_ and *t*_R(*trans*)_ are the retention times of the *cis*- respectively *trans*-analytes.

*ct*E_C_ = *t*_R(*cis*)_/*t*_R(*trans*)_ (4)

Again, based on the theory of the partition chromatography, the smaller retention times of *trans*-analytes compared to their *cis*-analytes would result from a weaker interaction strength of the *trans*-analytes with the GC stationary compared with the *cis*-analytes.

The author of the present article has hypothesized that the underlying mechanisms in *hd*IE_C_ and *ct*E_C_ are principally the same. These effects were examined by considering my own experimentally observed data for different classes of analytes and data published by other groups for unlabeled *cis*- and *trans*-fatty acids and for their ^2^H- and ^13^C-isotopologs [3,4,5,6]. As retention times of analytes were not available from many published articles (see Table 1) in order to determine *hd*IE_C_ and *ct*E_C_ values, only a small number of papers have been considered in the present work. All data used in the present study have been generated by validated GC-MS methods that included proper chemical derivatization of the analytes. The focus was on free amino acids and free fatty acids. Thus, amino acids (AAs) were analyzed as methyl ester-pentafluoropropionyl derivatives (AA-Me-PFP), and free fatty acids (FAs) either as methyl ester (FA-Me) or pentafluorobenzyl ester (FA-PFB) derivatives. The results of the present examination support the compiled hypothesis.

GC-MS and LC-MS are widely used in “classical” quantitative analyses and in metabolomics studies (Table 1). Nowadays, quantitative analyses are increasingly named targeted analyses. The expressions untargeted, nontargeted, non-targeted and semi-targeted analyses are widely used in metabolomics studies (Table 1), without a precise definition and often arbitrarily. The present work focused on quantitative GC-MS analyses, in which isotopologs had been used as internal standards. Chromatographic isotope effects and other types of chromatographic effects also occur in LC-MS and can even reach higher values. The majority of the studies considered in the present work are based on “classical” quantitative analyses using isotopologs and quadrupole-based, i.e., low-resolution mass spectrometers. Generally, chromatographic effects are independent of mass spectrometric resolution.

## 2. Methods

### 2.1. GC-MS Analyses Performed in the Author’s Group 

Analyses were performed by selected-ion monitoring (SIM) or selected-reaction monitoring (SRM) on a quadrupole (Q) type GC-MS and GC-MS/MS apparatus, namely TSQ 45, TSQ 7000 and ISQ (ThermoFisher, Dreieich, Germany). Fused silica capillary GC columns were used as specified below and in the Results section. Plasma, serum, and urine samples of human subjects had been collected and analyzed in previous peer-reviewed studies [7,8,9,10,11,12,13,14,15,16,17,18,19,20,21,22,23,24,25]. Helium and methane served as carrier and reagent gases for negative-ion chemical ionization (NICI), respectively. Aliquots (1 µL) of toluene, ethyl acetate or other extracts of analyte derivatives had been injected in the splitless mode. The concentrations of the stable-isotope labelled analogs (isotopologs) of the analytes added to the plasma and urine samples (at the start of the sample workup) were all relevant for the respective biological samples. 

Amino acids were analyzed as methyl ester pentafluoropropionyl (Me-PFP) derivatives using Optima 17 (15 m length, 0.25 mm i.d., and 0.25-µm film thickness) [26]. The oven used was kept at 40 °C for 0.5 min and ramped to 210 °C at a rate of 15 °C/min and then to 320 °C at a rate 35 °C/min. Commercially available isotopologs and in situ prepared trideutero-methyl esters of amino acids were used as internal standards.

Prostaglandin E_2_ (PGE_2_) was analyzed as a pentafluorobenzyl (PFB) ester methoxime (MO) trimethylsilyl (TMS) derivative (PFB-MO-TMS). Fatty acids and hydroxylated fatty acids were analyzed as PFB and PFB-TMS derivatives, respectively. Dimethylamine (DMA) was analyzed as a pentafluorobenzoyl derivative. Malondialdehyde (MDA) was analyzed as a di-PFB derivative. Information about the GC-MS methods of these and other analytes can be found in the References [7,8,9,10,11,12,13,14,15,16,17,18,19,20,21,22,23,24,25].

### 2.2. GC-MS Analyses Performed by Other Groups

The experimental conditions of the work performed by other authors are reported in the Results section. For better understanding, the nomenclature used in the original articles was retained. The original data and the newly calculated *hd*IE_C_ and *ct*E_C_ values are presented in the Results section of the present work in form of Tables and Figures as appropriate.

### 2.3. Calculations and Data Presentation

*hd*IE_C_ and *c*/*t*E_C_ values were calculated using Formulas (2) and (4), respectively. GraphPad Prism Version 7 for Windows (GraphPad Software, San Diego, CA, USA) was used for the statistical analyses and preparation of graphs. Chemical structures of the investigated analytes and their derivatives were drawn using ChemDraw 15.0 Professional (PerkinElmer Informatics, Germany).

## 3. Results

### 3.1. GC-MS Analysis of Miscellaneous Analytes—Author’s Group

The IE_C_ values for the PFB derivatives of ^14^N-nitrite, ^15^N-nitrite, ^14^N-nitrate, and ^15^N-nitrate in plasma and urine were close to 1.0000, with the difference in the retention times being not higher than 0.18 s [1].

Table 2 summarizes the GC-NICI-MS retention times of unlabeled (d_0_Me) amino acids (AAs) as methyl ester pentafluoropropionyl (AA-Me-PFP) derivatives, the chromatographic isotope effect (*hd*IE_C_) values of the AA-Me-PFP derivatives of unlabeled and deuterium labeled (d_3_Me) amino acid derivatives as measured simultaneously in urine samples of healthy humans. The retention times of all derivatives were measured with high precision (coefficient of variation). Under the same GC-MS conditions, very similar results were obtained from analyses of plasma samples of 38 healthy humans. The *hd*IE_C_ values of the AA-Me-PFP derivatives ranged between 1.002 and 1.006 (Table 2). 

**Figure 3:**
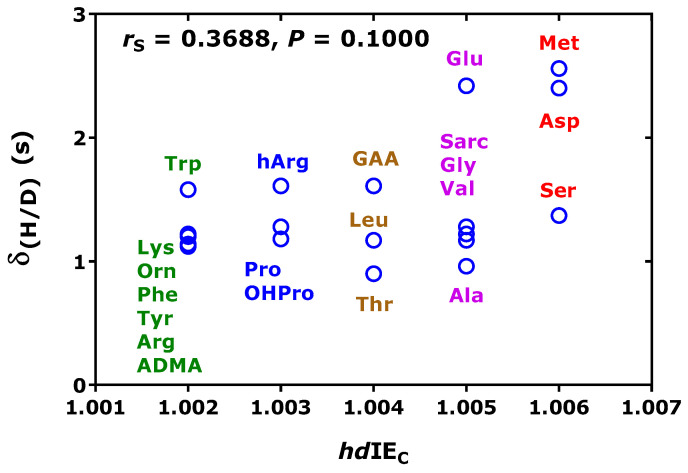
Relationship between the difference (δ_(H/D)_) in the retention times of unlabeled (AA-d_0_Me-PFP) and deuterated amino acids (AA-d_3_Me-PFP) and the chromatographic isotopic H/D effect (*hd*IE_C_). There is no correlation after Spearman between (δ_(H/D)_) and *hd*IE_C_. The Figure was constructed with the data listed in Table 2 using Formula ((3)).

Figure 3 shows that the difference (δ_(H/D)_) in the retention times of unlabeled (AA-d_0_Me-PFP) and deuterated amino acids (AA-d_3_Me-PFP) does not correlate with the chromatographic isotopic H/D effect (*hd*IE_C_). The greatest δ_(H/D)_ values were observed for glutamate (Glu), aspartate (Asp), and methionine (Met).

**Figure 4:**
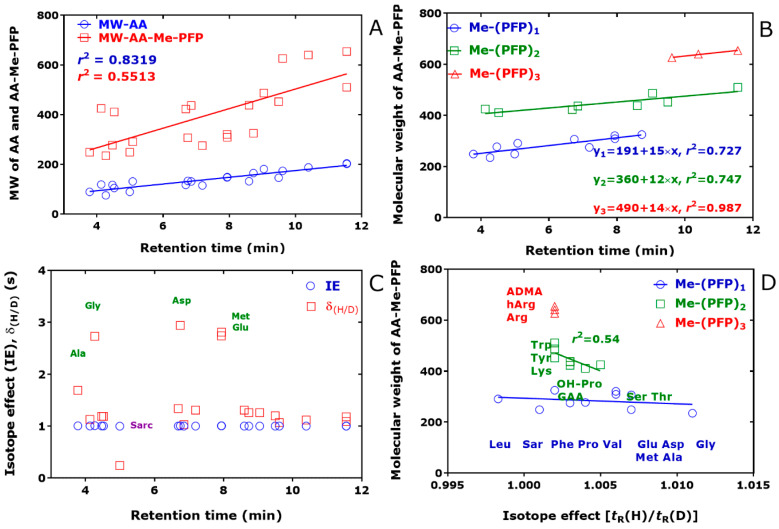
Relationship between the molecular weight (MW) of (**A**,**B**) non-derivatized amino acids (AA) and derivatized amino acids (AA-Me-PFP) and the retention time of the unlabeled amino acids AA-Me-PFP derivatives. (**C**) Isotope effect (IE, i.e., *hd*IE_C_) and difference (δ_(H/D)_) of the retention times of unlabeled (AA-d_0_Me-PFP) and deuterated amino acids (AA-d_3_Me-PFP) plotted versus the retention time of the unlabeled amino acids AA-Me-PFP derivatives. (**D**) Molecular weight of the AA-Me-PFP derivatives plotted versus *hd*IE_C_. The amino acids were analyzed in 29 human urine samples by GC-NICI-MS in the SIM mode as described previously [26] by using in situ prepared deuterated methyl esters of amino acids as internal standards. GC-MS apparatus, ISQ; GC column, Optima 17 GC column (15 m length, 0.25 mm i.d., and 0.25 µm film thickness). See also Table 2 and Figure 3.

Figure 4 shows the relationship between the molecular weight (MW) of native and derivatized amino acids in human urine and the retention time of the AA-Me-PFP derivatives. The retention time increased with increasing molecular weight of the non-derivatized amino acids (*r*^2^ = 0.83) and of their Me-PFP derivatives (*r*^2^ = 0.55). The weaker linearity of the Me-(PFP)_1_ and Me-(PFP)_2_ derivatives compared to the Me-(PFP)_3_ derivatives is presumably due to the combined effects of the structures of the side chain and of their derivatized amine and hydroxyl groups of the amino acids. Figure 4D suggests that the extent of the chromatographic isotope effect decreases with the decreasing molecular weight of the AA-Me-PFP derivative. 

**Figure 5:**
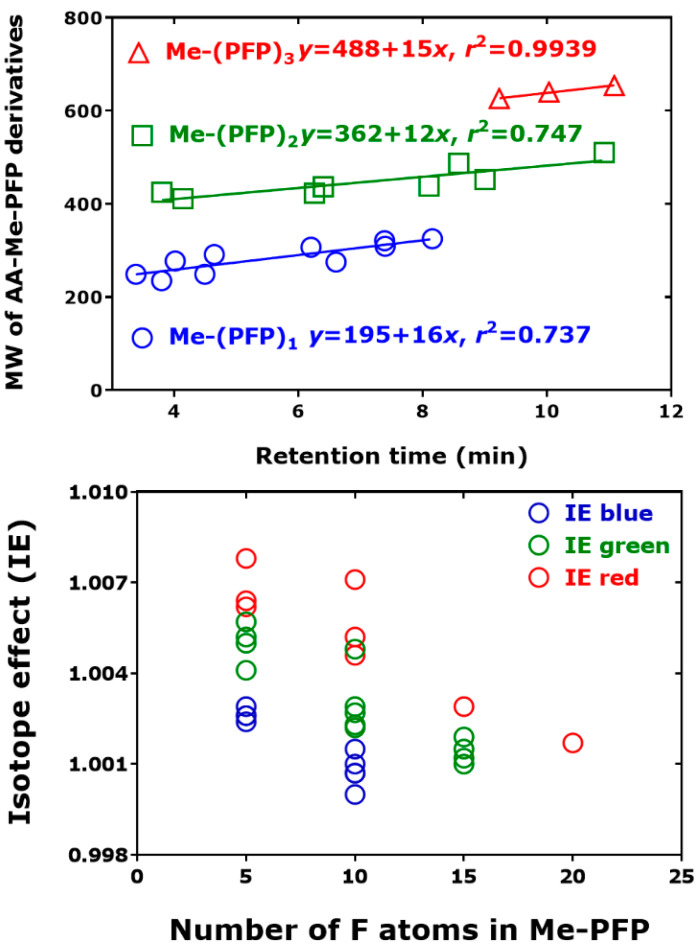
(***Upper panel***)*:* Molecular weight (MW) of the derivatized plasma amino acids (AA-Me-PFP) versus the retention time of the unlabeled amino acid AA-Me-PFP derivatives. (***Lower panel****):* Chromatographic isotope effect (IE, IE_C_) versus the number of the F atoms of the AA-Me-PFP derivatives. GC-NICI-MS in the SIM mode as described previously [26]. Symbol explanation for both panels: IE blue, Me-(PFP)_1_, 5 F atoms; IE green, Me-(PFP)_2_, 10 F atoms; IE red, Me-(PFP)_3_, 15 F atoms, and Me-(PFP)_4_, 20 F atoms.

Similar relationships were observed for the same amino acids analyzed in human plasma samples (Figure 5). The number of F atoms in the AA-Me-PFP derivatives decreases the chromatographic isotope effect: the bigger the number of the F atoms is, the weaker is *hd*IE_C_.

**Figure 6:**
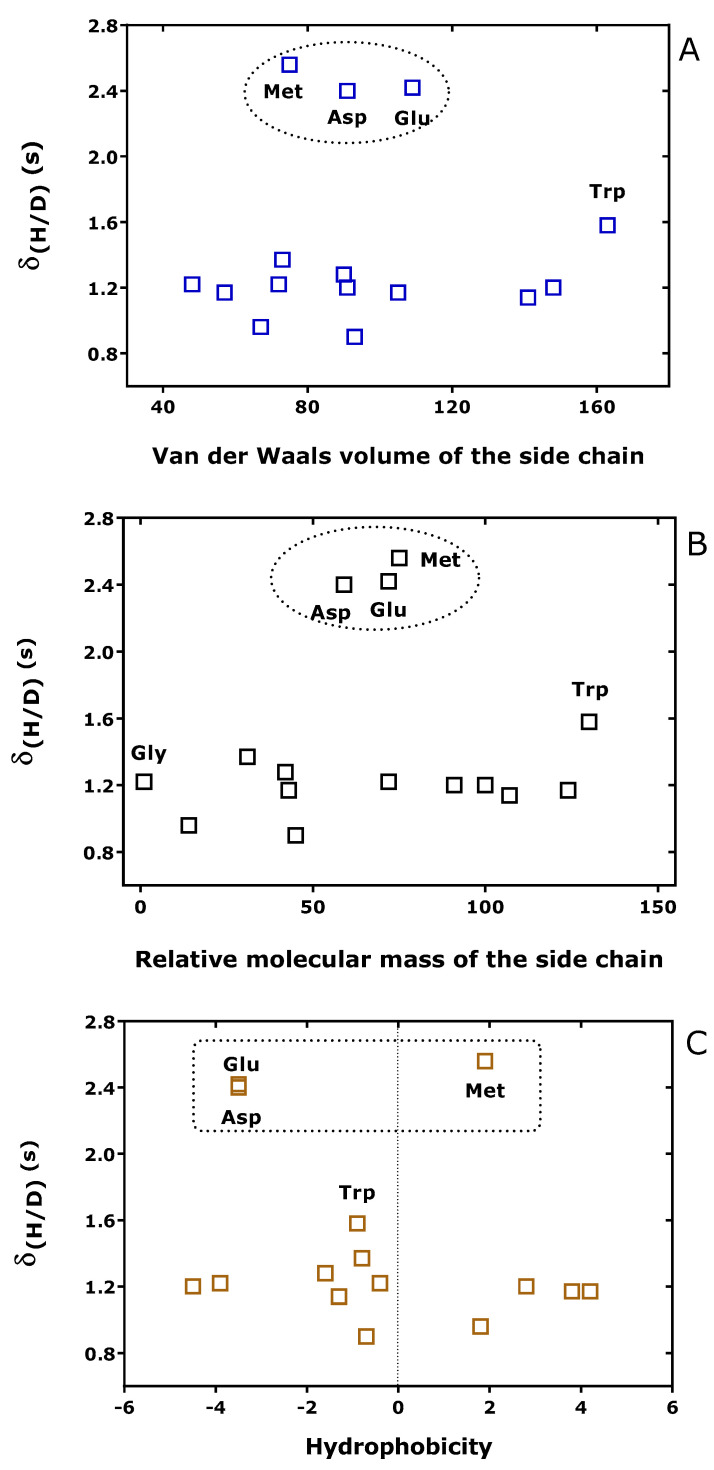
Difference (δ_(H/D)_) in the retention times of unlabeled (AA-d_0_Me-PFP) and deuterated amino acids (AA-d_3_Me-PFP) plotted versus (**A**) Van der Waals volume, (**B**) relative molecular mass (Gly = 1), and (**C**) hydrophobicity of the side chain of the unlabeled amino acids. GC-NICI-MS in the SIM mode was performed as described previously [26]. Literature data on van der Waals volume, relative molecular mass, and hydrophobicity of amino acids were used [27]. The Figure was constructed with the data listed in Table 1. The amino acid Glu, Asp, and Met are framed.

Figure 6 shows that δ_(H/D)_ is largely independent of the van der Waals volume, the relative molecular mass and the hydrophobicity of the unlabeled amino acids analyzed by GC-NICI-MS as Me-PFP derivatives. The greatest δ_(H/D)_ values were observed for Glu, Asp, and Met. Glu and Asp are dicarboxylic amino acids and form dimethyl ester. Met is a monocarboxylic sulfur-containing (thioether) amino acid. 

Table 3 compiles the calculated *hd*IE_C_ values observed from GC-NICI-MS and GC-NICI-MS/MS analyses of perfluorinated derivatives of biogenic amines, polyamines, and other physiological substances including creatinine. 

The *hd*IE_C_ values ranged between 0.9993 for the malondialdehyde (MDA) derivative, MDA-(PFB)_2_, and 1.0125 for the 5-hydroxyeicosanoic acid (HEA) derivative, HEA-PFP-TMS (Table 3). There was a positive correlation after Spearman (*r* = 0.7075, *p* = 0.0002) between the *hd*IE_C_ values and the number of D atoms in the derivatives of the analytes, but not between the *hd*IE_C_ and the MW values of the derivatives (*r* = 0.354, *p* = 0.106). Yet, *hd*IE_C_ and MW of the native, non-derivatized analytes correlated with each other (*r* = 0.499, *p* = 0.018). These results suggest that the PFB, PFBz, and PFP residues, which were introduced by the derivatization of the analytes, decrease the extent of the *hd*IE_C_. 

### 3.2. hdIE_C_ in the GC-MS/MS Analysis of Fatty Acid Methyl Esters—Tintrop et al. 2022, 2023 [5,6]

Fatty acids (FAs) occur in biological samples in free form (FFA) and as methyl esters (FAMEs). The simultaneous GC-EI-MS/MS determination of FAs and FAMEs was reported [5,6]. This method includes solvent-free solid-phase microextraction arrow headspace extraction and in situ isotope-labeling of FFAs with deuterated methanol (CD_3_OD). It utilizes the chromatographic isotope effect and the 3 Da-shift due to d_3_Me compared to d_0_Me. Sulfuric acid-catalyzed derivatization of FFAs with CD_3_OD was performed for 20 min with 4 vol% CD_3_OD and a pH value of 2.1.

The simultaneous analysis of FAMEs was performed on a GC 2010 with a MS/MS TQ8040 (Shimadzu Deutschland GmbH, Duisburg, Germany) in the MRM (SRM) mode. A Zebron ZB-FAME capillary column (30 m × 0.25 mm × 0.20 µm, Phenomenex, Torrance, USA) was used. The oven temperature program started at 40 °C, which was kept for 5 min, and then raised with a rate of 5 °C/min to 210 °C, where it was held for 5 min. Helium was used as a carrier gas (1.8 mL/min) and argon as a collision gas. Injection of the analytes was performed by splitless thermal desorption. 

**Figure 7:**
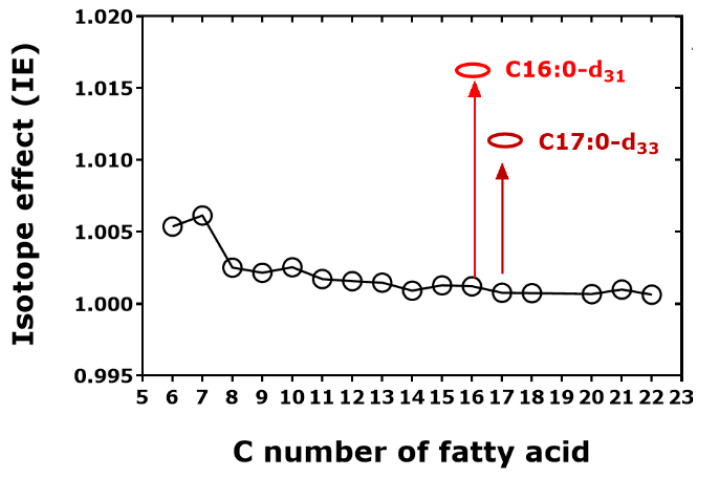
*hd*IE_C_ values versus the number of the C atoms in unlabeled saturated and unsaturated fatty acid methyl esters (FA-d_0_Me) and deuterium-labeled saturated and unsaturated fatty acid methyl ester (FA-d_3_Me). Inserts indicate the *hd*IE_C_ values for the unlabeled methyl esters of C16:0-d_31_ and C17:0-d_33_. The Figure was constructed with data reported by Tintrop et al. 2023 [6].

The investigated fatty acids included saturated (C6:0 to C22:0) and unsaturated C16 and C18 fatty acids, as well as perdeuterated C16:0, i.e., C16:0-d_31_, and C17:0, i.e., C17:0-d_33_. The retention times of the FA-d_0_Me and FA-d_3_Me derivatives reported by the authors [6] were used to calculate the *hd*IE_C_ values. The newly calculated data are summarized in Table 4 and illustrated in Figure 7. The retention time of the FA-Me derivatives did not increase linearly with the number of the C-atoms of the native fatty acids when considering all fatty acids (*r*^2^ = 0.9813).

The H/D isotope effect is evident. Expectedly, the strongest chromatographic isotope effects are seen for the methyl esters of the perdeuterated fatty acids C16:0-d_31_ and C17:0-d_33_. The IE_C_ values decrease with the increasing length of the fatty acid molecule. These observations suggest that the entire H/D effect of the d_3_Me group decreases with the increasing length of the fatty acid molecules by a factor of 10 (from 0.6% to 0.06% with respect to the retention time). According to the manufacturer’s information, Zebron™ ZB-FAME is based on a high-cyanopropyl (G48) chemistry (O-Si-(C_3_H_6_CN)*_n_*) and has high polarity. Analogous to the isotope effect seen in the GC-MS analysis of the methyl esters of amino derivatives, one may assume that the deuterium atoms of the d_3_Me ester group of the fatty acids weaken the interaction of the lipophilic fatty acid molecules with the immobilized liquid phase of the GC column. This effect is maximum in C16:0-d_31_ (IE_C_, 1.0171) and C17:0-d_33_ (*hd*IE_C_, 1.0126). The *ct*E_C_ values for C18:1c and C18:1t are calculated to be each 1.0041 for d_0_Me and d_3_Me, i.e., they are higher than the *hd*IE_C_ values of 1.0011.

Similar effects have been reported by Alexander et al. [28] for 45 unlabeled *cis/trans* fatty acid methyl esters using a highly polar cyanopropylsiloxane SP 2560 fused-silica capillary column (100 m × 0.25 mm × 0.20 µm). The GC column oven temperature was linearly programmed from 80 °C to 220 °C at 8 °C/min. Initial and final temperatures were held for 2 min and 32 min, respectively, with a total run time of 55 min, using helium as the carrier gas (0.67 mL/min). For example, the retention times were reported to be 35.7 min for C22:0, 37.2 min for C22:1, 44.6 min for C22:4, 46.5 min for C22:5, and 52.0 min for C22:6.

Ecker et al. [29] used a highly polar BPX70 GC column (10 m × 0.10 mm × 0.20 µm; 70% cyanopropyl polysilphenyl-siloxane) for the GC-MS analysis of *cis-* and *trans*-fatty acid methyl esters. The initial oven temperature was held for 0.75 min, then programmed to increase with 40 °/min to 155 °C, with 6 °C/min to 210 °C, to finally reach 250 °C with 15 °C/min, which was held for 2 min. FAMEs eluted between 2.54 min (C8:0) and 14.16 min (C28:0). Unsaturated FAMEs had higher retention times than saturated FAMEs. The *ct*E_C_ values were 1.0154 for C18:1 (c9) and C18:1 (t9), and 1.0364 for C18:2 (c9,c12) and C18:2 (t9,t12) [29]. Within unsaturated FAMEs, the retention time was higher when the –C=C-double bonds were near to the terminal methyl group of the fatty acids.

### 3.3. hdIE_C_ in the GC-MS Analysis of Fatty Acid-Pentafluorobenzyl Esters—Quehenberger et al. 2011 [4]

Unlabeled and deuterium-labeled FAs (Table 5) were analyzed by GC-NICI-MS as PFB ester derivatives. GC-NICI-MS analysis was carried out on an Agilent 6890N gas chromatograph equipped with an Agilent 7683 autosampler (Santa Clara, CA, USA) using an Agilent 5973 mass selective detector. The FA-PFB esters dissolved in isooctane were injected with a pulsed (25 psi) splitless injection mode onto a Zebron ZB-1 column (15 m × 0.25 mm i.d., coated with 100% dimethylpolysiloxane; Phenomenex, Torrance, CA, USA). Helium (0.9 mL/min) was used as a carrier gas. The GC oven temperature was programmed from 150 °C to 270 °C at 10 °C/min, ramped to 240 °C at 40 °C/min and held at 240 °C for 1 min. The injector and the transfer line were kept at 250 °C and 280 °C, respectively. Methane was used as the reagent gas for NICI at an ion-source temperature of 150 °C. SIM of the [M-PFB]^−^ anions of the FA-PFB esters was performed. The [M-PFB]^−^ ions correspond to the carboxylate anions.

**Figure 8:**
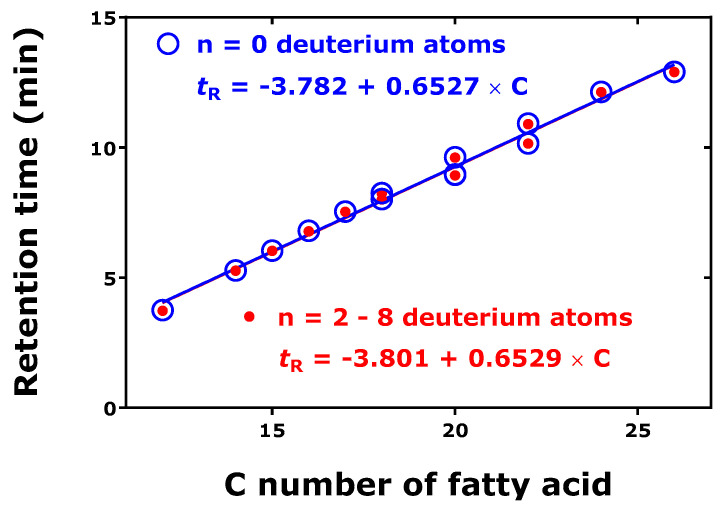
Relationship between the retention time and the number of C atoms in the fatty acids of the unlabeled and deuterium-labeled FA-PFB esters. Inserts indicate the linear regression equations. The Figure was constructed with data reported by Quehenberger et al. 2011 [4]. See also Table 5.

The retention time increased linearly with the increasing number of C-atoms of the native fatty acids (Figure 8). The highest *hd*IE_C_ value was observed for the shortest fatty acid (i.e., C12:0), and the lowest *hd*IE_C_ value for the longest fatty acids (i.e., C24:0 and C26:0). There was no dependency of the *hd*IE_C_ value upon the number of the deuterium atoms in the fatty acid molecules. 

These observations suggest that the H/D isotope effect is evident in the GC-MS analysis of the FA-PFB ester derivatives. However, the isotope effect is weak. A possible explanation could be that the interaction of the PFB ester residue of the fatty acid derivatives with the stationary phase outweighs the interaction of the H/D atoms of the fatty acid skeleton.

### 3.4. Isotope Effects in the GC-MS Analysis of Cis- and Trans-Fatty Acid Pentafluorobenzyl Esters—Kuiper et al. 2018 [3]

Kuiper and colleagues developed a GC-NICI-MS method for the quantitative analysis of *trans*-fatty acids in human plasma, serum, and red blood cells (RBCs). As the *trans*-fatty acids coexist in biological samples at considerably lower concentrations, a “special” GC column known to allow simultaneous analysis of *trans*- and *cis*-fatty acids was used, i.e., an Agilent Select FAME.

Samples (100 µL) were combined with 100 μL of an internal standard (IS) solution that contained isotopologs. Subsequently, the samples were hydrolyzed (2 mL of 10% *v/v* 6 M HCl in acetonitrile followed by 2 mL of 10% *v/v* 10 M NaOH in methanol), each carried out at 104 °C for 45 min. After neutralization with 6 M HCl, the free FAs were extracted with hexane (three times, 2 mL each). The solvent was removed under vacuum (Genevac, Stone Ridge, NY, USA) and the samples were derivatized at room temperature for 15 min with 100 μL of 7% PFB-Br in acetonitrile and 10 μL triethylamine as the base catalyst. The fatty acid PFB (FA-PFB) esters were extracted with hexane (500 μL) and transferred to autosampler vials for GC-MS analysis. Samples were handled in glass vials to minimize contamination of samples with FAs from plastic supplies.

GC-MS analysis of 27 FAs and 18 ISs was carried out on a 7890/5975C GC–MSD from Agilent Technologies (Santa Clara, CA, USA). The inlet temperature was 240 °C and 1-μL aliquots were injected with a 100:1 split ratio using a Gerstel Multipurpose Sampler MPS (Gerstel, Mülheim an der Ruhr, Germany) equipped with a cool drawer set at 10 °C. The carrier gas was hydrogen at a flow rate of 2 mL/min. An Agilent Select FAME (200 m × 250 μm × 0.25 μm) GC column on a cyanopropyl basis (CP-7421) was used. The column temperature was ramped from 50 °C to 160 °C at 40 °C/min, held at 160 °C for 10 min, increased by 1 °C/min to 175 °C, then by 0.5 °C/min to 210 °C, and finally by 35 °C/min to 260 °C, where it was held for 25 min. NICI was performed using methane as the reagent gas. The ions [M-PFB]^−^ were used in the SIM mode (Table 6). The transfer line, source, and quadrupole temperatures were 260 °C, 230 °C, and 150 °C, respectively. The electron multiplier voltage was adjusted throughout the chromatographic run to increase sensitivity for lowabundance FAs and prevent detector saturation for high-abundance FAs.

The fatty acids analyzed and the *m/z* values of the ions [M-PFB]^−^ used in GC-NICI-MS analyses are reported in Table 6. This Table also summarizes the retention times of the FA-PFB derivatives obtained from the GC-NICI-MS analyses of the indicated *trans*-fatty acids (*t*), the regular fatty acids including the *cis*-fatty acids (*c*), and their ^2^H- and ^13^C-isotopologs. The retention times reported by Kuiper et al. [3] were used in the present work to newly calculate the *cis*-FA-PFB-to *trans*-FA-PFB ratios (c/t) and the *hd*IE_C_ values, as applicable. 

There were three *cis/trans*-FA pairs. The FA-PFB derivatives of the *cis*-fatty acids had longer retention times than the corresponding *trans*-fatty acids. The *c/t* values amounted to 1.0233, 1.0171, and 1.0163 (Table 6). The retention times of the FA-PFB derivatives of the *trans*-fatty acids and their corresponding ^13^C-isotopes were almost identical, indicating no chromatographic isotope effect due to ^13^C/^12^C. The FA-PFB derivatives of di-unsaturated fatty acids had higher retention times than their respective mono-unsaturated fatty acids. Note the considerably longer retention times compared to those in the previous studies (Table 4, Table 5 and Table 6).

There are nine pairs of FAs and their ^2^H-labeled isotopologs. The FA-PFB derivatives of the unlabeled fatty acids had longer retention times than their ^2^H-isotopologs. The calculated *hd*IE_C_ values ranged between 1.0428 for myristic acid (C14:0 and D_27_-C14:0) and 1.0009 for arachidonic acid (C20:4n-6,9,12,15 and D_8_-C20:4n-6,9,12,15).

These observations suggest that the FA-PFB ester derivatives of *trans*-fatty acids behave towards their *cis*-fatty acids in the same manner as behave non-deuterated towards deuterated fatty acids with respect to gas chromatography in fused-silica capillary columns. Note the long retention time range of the investigated FA-PFB ester derivatives in the study (i.e., 56 min to 116 min).

**Figure 9:**
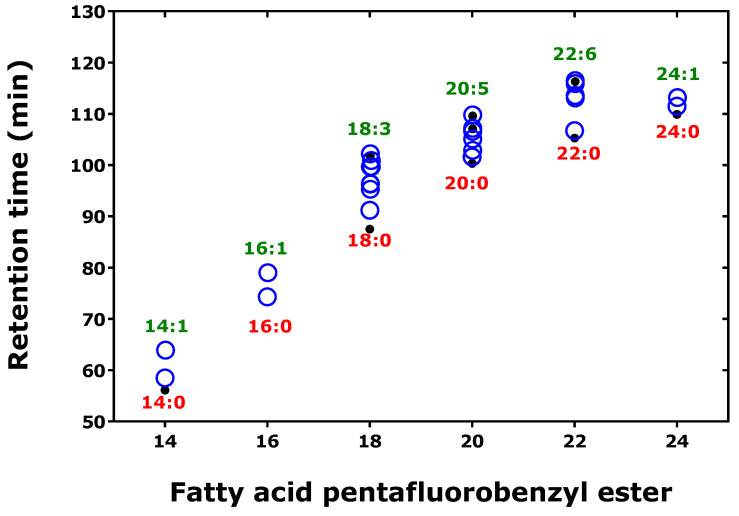
Retention times of the pentafluorobenzyl ester derivatives of the indicated saturated and non-saturated fatty acids and the number of the C atoms in the native fatty acids analyzed as pentafluorobenzyl esters. Open symbols, unlabeled and ^13^C-labeled fatty acids; closed symbols, deuterium labeled fatty acids. The Figure was constructed with data reported by Kuiper et al. 2018 [3]. See also Table 7.

A further interesting observation is that the retention times of the FA-PFB ester derivatives increased with the increasing grade of unsaturation of fatty acids, e.g., within C_18_- and C_20_-fatty acids (Figure 9, Table 6 and Table 7), suggesting enhancement of interaction between the immobilized stationary phase of the GC column and the backbone of the fatty acids. In that study, the retention times of the FA-PFB ester derivatives did not increase linearly with the number of the C-atoms of the native fatty acids (Figure 9 vs. Figure 8). 

The median concentrations of *trans*-fatty acids in plasma, serum, and RBCs of 66 healthy donors ranged between 1.8 µM and 2.1 µM for C16:1n-7 t, 6.2 µM and 8.1 µM for C18:1n-9 t, 8.8 µM and 11.4 µM for C18:1n-7 t, and 0.6 µM and 1.0 µM for C18:2n-6 t,9 t [3]. These concentrations account for 0.01–0.10% of total fatty acids in blood.

## 4. Discussion

Chromatographic separation of derivatized analytes in GC-MS is based on continuously occurring interactions of the analyte derivatives between a mostly silicone-based stationary phase (of small inner diameter and film-thickness) immobilized inside the GC column and mostly helium as the mobile phase. The just now “free“ analyte derivatives are “carried” by helium through the GC column until they are released into the ion-source of the GC-MS apparatus. The physicochemical properties of the analytes (e.g., boiling point, molecular weight, chemical structure, and possibly changing shape and orientation in the gas phase) contribute to the chromatographic effects, which are finally manifested in the retention times of the analytes. The retention time of analytes is a useful measure to quantify chromatographic effects. In quantitative GC-MS analyses, stable-isotope labeled analytes are used as internal standards.

Kinetic isotope effects, chromatographic isotope effects, and chromatographic *cis/trans*-effects have been known for several decades, but the underlying mechanisms are incompletely understood. Chromatographic isotope effects (IE_C_s) are assumed to originate from differences in physicochemical properties of unlabeled and stable-isotope labeled analytes that result from the differences introduced into the analytes mainly by the heavy isotopes of H (*hd*IE_C_), C, N and O, i.e., ^2^H (D), ^13^C and ^15^N, respectively. Perhaps easier to understand are the chromatographic *cis/trans*-effects (*ct*E_C_), as the orientation of the molecules in the space may differ considerably, for instance in dependence on the residues in olefinic analytes R_1_-C=C-R_2_.

The retention time (*t*_R_) in gas chromatography (GC) and liquid chromatography (LC) is an experimentally ascertainable integral parameter, which incorporates all factors that are involved in the chromatographic process. In GC, they include boiling points vapor pressure, chemical composition, and three-dimensional structure of the analyte derivatives, the chemical composition of the stationary phase, adsorption/desorption processes, temperature of the injector port, initial and subsequently increasing temperature of the GC column and its dimensions (length, inner diameter, film thickness), as well as the nature and flow rate of the carrier gas. The present work addressed the issue of *hd*IE_C_ and *c*tE_C_ effects_,_ and investigated these phenomena on a quantitative basis by re-examining data reported in the literature mainly by four groups including the author’s group. We hypothesized that the chromatographic isotope- and *cis/trans*-effects can be quantified by using the retention times of analyte derivatives in GC and introduced the parameters *hd*IE_C_ and *ct*E_C_. The *hd*IE_C_ and *c*tE_C_ values were calculated by Formulas (2) and (4), respectively. The main working hypothesis was that both effects can be explained by differences in the interaction of the analytes with the stationary phase of fused-silica capillary GC columns. One class of analytes were amino acids that were analyzed as methyl ester pentafluoropropionic (PFP) derivatives. The second class free fatty acids that were analyzed as methyl ester (Me) or pentafluorobenzyl ester (PFB) derivatives. 

The utilized GC columns differed in the chemistry of the stationary phase, and in part in length (15 m, 30 m, 200 m), had a comparable internal diameter, and small differences in film thickness(0.20 µm and 0.25 µm) of the immobilized stationary phase. The volume of the GC lumen was calculated to be about 0.7 mL, 1.5 mL and 9.6 mL, respectively. Under consideration of the reported carrier gas, and presumably laminar than turbulent flow, it is calculated that the carrier gas would need about 0.7 min to 5 min for a passage through the GC columns. The shape of the analytes inside the GC columns is unknown. In the case of hydrophobic analyte derivatives such as the FA-PFB derivatives, oblongness could possibly prevail. The interaction of the analyte derivatives and the stationary phase is considered to be due to different forces including van der Waals forces. Other effects, such as the organic solvent extract used in GC-MS analyses, “condensation” of analyte derivatives at lower GC column temperatures, and competition between the derivatives of analytes and their isotopologs, are possible but were not considered in this work. 

Schmarr et al. [30] and Thakur et al. [31] reported on normal and inverse chromatographic isotope effects for various analytes using different stationary phases. In the case of normal isotopic effects, deuterated analytes elute later than the protiated analogs. In the case of inverse isotopic effects, deuterated analytes elute earlier than the protiated analogs. Thus, the observations described in the present work can be categorized as inverse isotopic effects. Schmarr et al. [30] and Thakur et al. [31] did not report retention times of the isotopologs pairs so that the *hd*IE_C_ values could not be reported in this article. It was observed that nonpolar stationary phases often exhibited an inverse isotope effect, whereas polar stationary phases often showed a normal isotope effect. For instance, by using the stationary phase SPB-20 (bonded; poly(20% diphenyl/80% dimethyl siloxane) phase; polarity number, 111), C_6_D_4_-(CD_3_)_2_ (*o*-xylene-d_10_) eluted in front of C_6_H_4_-(CH_3_)_2_ (*o*-xylene-d_0_) with a peak resolution of 2.70 (complete baseline separation, i.e., inverse *hd*IE_C_). By using the stationary phase IL111i (non-bonded; 1,5-di(2,3-dimethylimidazolium)pentane bis(trifluoromethanesulfonyl)imide phase; polarity number, 12), C_6_D_4_-(CD_3_)_2_ (*o*-xylene-d_10_) eluted behind C_6_H_4_-(CH_3_)_2_ (*o*-xylene-d_0_) with a peak resolution of 1.00 (i.e., normal *hd*IE_C_). All analytes investigated with the stationary phase IL111i were found to be associated with normal *hd*IE_C_ [31]. The stationary phase IL111i was found to be the only stationary phase that interacts with molecules by π–π, dipole–dipole, and dipole–induced dipole interactions in addition to typical hydrogen bonding, dispersive, and acid-base type interactions [31].

### 4.1. Chromatographic Isotope Effects

The IE_C_ values observed for PFB-O*NO_2_ and PFB-*NO_2_ (an asterisk * indicates ^14^N/^15^N; difference of 7% in atom mass) were very close to 1.0000 and indicate lack of a measurable IE_C_ effect and virtually the same interaction extent of the *central* ^14^N and ^15^N atoms of PFB-O*NO_2_ and PFB-*NO_2_ due to sterical hindrance through the neighboring C and O atoms.

The IE_C_ values observed for the ^13^C isotopologs of the fatty acid derivatives are almost identical to those of the ^12^C isotopologs, while the simultaneously analyzed ^2^H isotopologs clearly caused IE_C_. The C atoms of analytes do not interact directly with the stationary phase of the GC column. Furthermore, the ^13^C atoms of the ^13^C-labeled analytes differ by only 8% in atom mass from the ^12^C atoms. These two factors are likely responsible for the missing isotope effect in the ^13^C-labeled fatty acid derivatives.

The two *N*-methyl groups of dimethylamine (DMA) and metformin (Metf) in their PFBz, respectively, PFP derivatives caused strong *hd*IE_C_ effects due to the six H/D atoms of the methyl groups which are not hindered sterically in their interaction with the stationary phase of the GC column. The comparably stronger *hd*IE_C_ effects are likely to result from the greater differences in the mass of the H and D atoms (100% increase in atom mass).

In agreement with the observations presented in the Results section, Benchekroun et al. [32] reported that analytes with CD_3_ groups influence the *hd*IE_C_ of derivatives of caffeine and of its metabolites obtained on a 14% cyanopropylphenyl)methylpolysiloxane fused-silica capillary column in GC-EI-MS (EI, electron ionization). The *hd*IE_C_ (range, 1.0012 to 1.0045) was also found to increase with the number of the D atoms and their position in the molecules [32]. 

The CODATA-recommended value for the charge radius of D is 2.5 times bigger than the charge radius of H: 2.13 fm vs. 0.84 fm (https://physics.nist.gov/cuu/Constants/index.html; accessed on 10 December 2024). The Van der Waals radius is 120 pm for H, 135 pm for F, 152 pm for O, and 160 pm for C. The rate of a reaction involving a C–H bond is typically 6–10 times faster than the corresponding C–D bond, meaning that the heavier atom favors a stronger bond [33,34]. These two physicochemical factors are likely to be dominant contributors to the chromatographic isotope effects observed in the present study, with the van der Waals forces being more relevant for fatty acids [35], but not for amino acids. 

The bigger charge radius of the D atom lends to the deuterated analytes larger molecular volumes compared to the protiated analytes. At the same time, the stronger C-D bonds tend to interact more weakly with the stationary phase of GC columns such as those made of 50% methylpolysiloxane/50% phenylpolysiloxane or 14% (cyanopropylphenyl)methylpolysiloxane. The synergetic effects of these factors is supported by the highest *hd*IE_C_ values observed for the perdeuterated fatty acids. Other non-deuterated voluminous structures of the analytes such as in the PFB esters are likely to attenuate the *hd*IE_C_ [4].

It is reasonable to assume that deuterated analytes may have lower boiling points and higher volatilities analogous to organic solvents such as acetone and benzene, because of stronger intra-molecular compared to inter-molecular interactions. Similar differences in the boiling points also prevail in perfluorinated chemicals including toluene (Bp of CH_3_-C_6_H_5_, 110.5 °C; Bp of CF_3_-C_6_F_5_, 104 °C), which would result in H/F-related effects (*bp*E_H/F_) of 1.0625. The *bp*E_H/F_ values for PFB-Br and pentafluoropropionic anhydride (PFPA) used for the derivatization and GC-MS analysis of fatty acids, respectively, amino acids are calculated by Formula (5) to be 1.1375 and 2.3857. Thus, it can be reasonably expected that the physicochemical properties of the PFB and PFP residues are transferred to the analyte derivatives.

*bp*E_H/F_ = Bp_(H)_/Bp_(F)_ (5)

whereas Bp_(H)_ and Bp_(F)_ are the boiling points of the protiated and perfluorinated solvent, respectively.

The *hd*IE_C_ values observed in the present work may have been influenced by different experimental parameters, mainly including the length and chemistry of the GC columns used, the starting GC oven temperature and the GC oven temperature gradient. Thus, the starting oven temperature was 150 °C in the GC-MS analysis of the FA-PFB esters by Quehenberger et al. [4]), but only 40 °C in the GC-MS analysis of the FA-Me esters by Tintrop et al. [6]. At (very) high starting GC oven temperatures and high GC oven temperature gradients, chromatographic separation may be inadequate for studying chromatographic isotope effects.

### 4.2. Chromatographic cis/trans-Effects in Fatty Acid Derivatives

**Figure 10:**
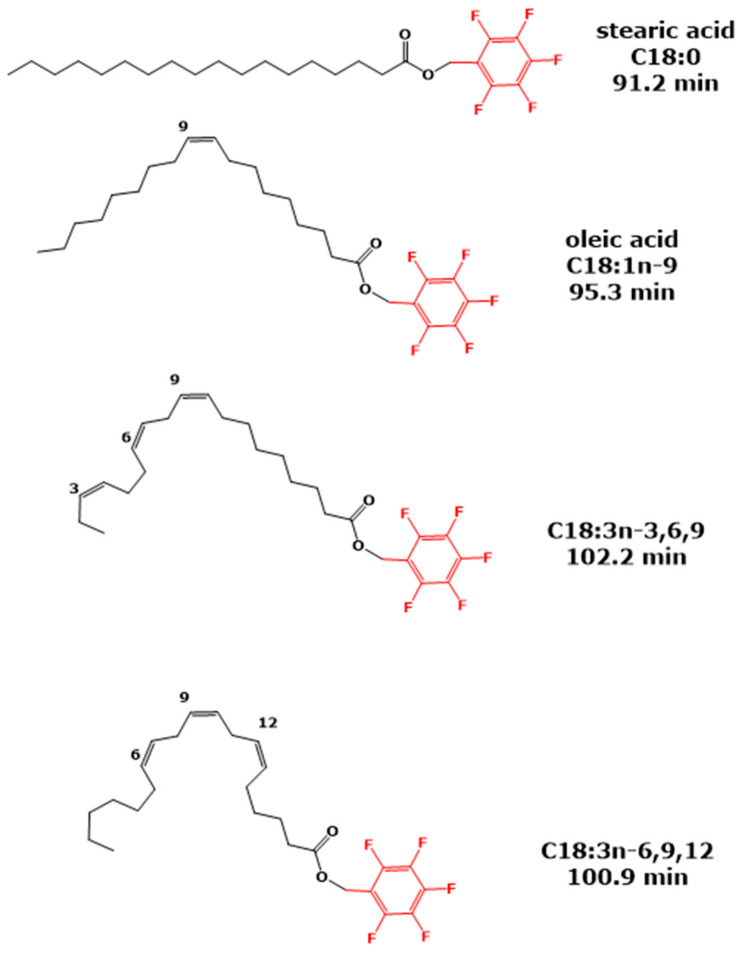
Chemical structures of the PFB ester derivatives of the saturated stearic acid (C18:0), the mono-unsaturated *cis*-oleic acid (C18:1n-9) and two isomers of *cis*-linolenic acid, i.e., α-linolenic acid (C18:3n-3,6,9) and γ-linolenic acid C18:3n-6,9,12), and their retention times reported by Kuiper et al. 2018 [3]. See also Table 7.

Many of the issues discussed above on the chromatographic isotope effects can be translated to the chromatographic *cis/trans-*effects. Possibly the greatest difference between IE_C_ and *c/*tE_C_ refers to the “interaction surface” of the analytes due to the *cis/trans* isomerism as observed in the case of unsaturated fatty acids. The *cis/trans* configuration “subdivides” the analyte derivative, depending on the position of –C=C-double bond(s), into two or more *cis*- and *trans*-oriented structures in the lumen of the GC column. The *trans*-isomers probably do not to interact with the GC stationary phase to the same degree as the *cis*-isomers, because the *cis*-configuration allows for a stronger interaction of the –C=C-double bonds with the stationary phase. This is schematically illustrated for the PFB ester derivatives of the saturated stearic acid (C18:0) and the unsaturated *cis*-fatty acids oleic acid (C18:1), α-linolenic acid and γ-linolenic acid (both C18:3) (Figure 10). 

The shape of the FA-PFB derivatives could be elongated for saturated fatty acids but curvy for unsaturated *cis*-fatty acids. Curvy fatty acids may possibly collide more often with the GC stationary phase through their –C=C-double bonds, thus increasing the residence time in the lumen of the GC column.

### 4.3. Chromatographic Effects Due to Other Types of Isomerism

Similar to the chromatographic *cis/trans*-isomerism in unsaturated fatty acids is the stereo-isomerism of isobaric prostaglandins (PG). The most commonly analyzed isomeric prostaglandins are the F_2_-isoprostanes (four types, 64 isomers) [36]. The best investigated F_2_-isoprostanes are prostaglandin F_2α_ (PGF_2α_, 9α,11α,15*S*-trihydroxy-(**8α**)-prosta-5*Z*,13*E*-dien-1-oic-acid) and 8-*iso*-prostaglandin F_2α_ (8-*iso*-PGF_2α_,9α,11α,15*S*-trihydroxy-(**8β**)-prosta-5Z,13*E*-dien-1-oic-acid), and prostaglandin E_2_ (PGE_2,_9-oxo-11α,15*S*-dihydroxy-(**8α**)-prosta-5*Z*,13*E*-dien-1-oic acid) and 8-*iso*-prostaglandin E_2_ (8-*iso*-PGE_2_,9-oxo-11α,15S-dihydroxy-(**8β**)-prosta-5*Z*,13*E*-dien-1-oic acid) [23,37]. The two epimers have an opposite configuration at only one stereogenic center out of at least two. PGF_2α_ and 8-*iso*-PGF_2α_ differ in the space orientation of the α-chain on the cyclopentane C-8 atom in the molecules. The PFB-TMS derivatives of PGF_2α_ and 8-*iso*-PGF_2α_ behave like a *cis/trans* isomer with respect to the plane of the cyclopentane ring. The retention time of PGF_2α_-PFB-(TMS)_3_ in GC-MS/MS was determined to be 23.70 min and that of 8-*iso*-PGF_2α_-PFB-(TMS)_3_ 22.97 min [37], resulting in a retention time ratio of 1.0318. This value is even greater than the *c/t*E_C_ values of *cis/trans* FA-PFB derivatives. No such effect was observed for the PFB-MO-(TMS)_2_ derivatives of PGE_2_ and 8-*iso*-PGE_2_, but it occurred in the bigger pentafluorobenzyloxime (PFBO) derivatives, i.e., PFB-PFBO-(TMS)_2_ derivatives [23]. *hd*IE_C_ effects of similar extent were observed in both prostaglandins [23,37]. This effect could be named the chromatographic epimer effect *epi*E_C_, and the underlying mechanism is assumed to be the same as the *ct*Ec.

Isomerism is not limited to endogenous substances, but it also applies to synthetic compounds such as the polychlorinated terphenyls (PCTs), which include *ortho*-, *meta*- and *para*-homologs. In GC-MS (DB-5 column, (5%-phenyl)-methylpolysiloxane), the elution order of isomers was found to be *ortho*-PCTs, *meta*-PCTs and *para*-PCTs [38], obviously in the order of increasing melting points (58 °C, 86 °C, 212 °C, respectively) and boiling points (337 °C, 379 °C, 389 °C, respectively). Similar effects have been observed by GC-MS (Elite–624 capillary column; 6% cyanopropyl phenyl 94% methyl, 30 m × 0.25 mm id, 1.4 µm film-thickness) for the xylene isomers *ortho*-xylene, *meta*-xylene and *para*-xylene, with the *meta*-xylene and *para*-xylene being more closely and earlier eluting than *ortho*-xylene (*t*_R_: 12.2 min, 12.00 min, 12.98 min, respectively; Bp: 139 °C, 137 °C, 144 °C, respectively) [39]. In the case of the *o/m/p*-isomers, the position of the two methyl groups and the size of the aromatic ring available for interaction with the stationary phase of the GC column are likely to influence more strongly the retention time of the xylene isomers. In the case of the *o/m/p*-isomers of the polychlorinated terphenyls, the surface of the three aromatic rings of the longish *para*-PCTs isomers is likely to be more strongly exposed to the stationary phase of the GC column than that of *ortho*- and *meta*-PCTs isomers.

In GC-MS, GC separation depends on the relative strength of the analytes–stationary phase interaction. It was calculated that the orientation polarization of *meta*-xylene is 80 times greater than of *para*-xylene, and that the permanent dipole moment of *meta*-xylene is 15 times greater than of *para*-xylene [22]. Kanai and colleagues assumed that compared with *para*-xylene, *meta*-xylene has a greater Debye–Keesom (both belong to the van der Waals forces) interaction tendency with the polar polyethylene glycol (PEG) capillary column [40].

### 4.4. Sterical Effects on Chromatographic Isotope Effects

**Figure 11:**
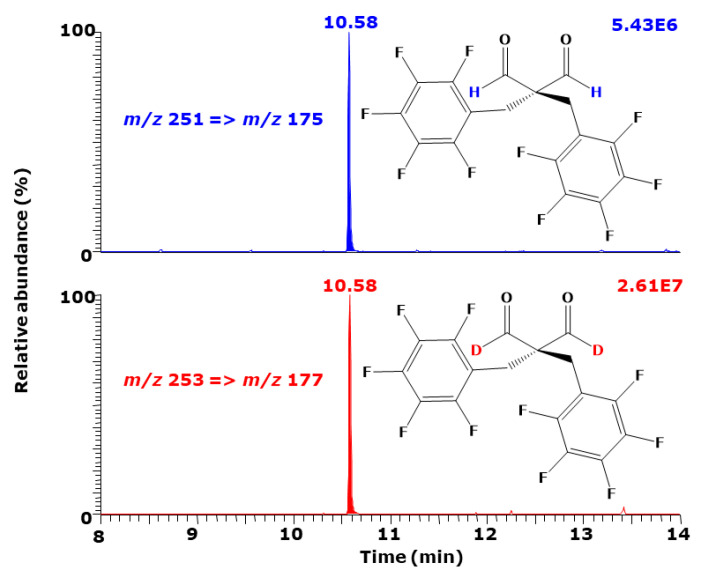
GC-MS/MS chromatogram from the analysis of malondialdehyde and [1,3-^2^H_2_] malondialdehyde as pentafluorobenzyl derivative. SRM of the mass transition *m/z* 251 → *m/z* 175 for O**H**C-C(PFP)_2_-C**H**O (*t*_R_, 10.58 min) and *m/z* 253 → *m/z* 177 for O**^2^HC**-C(PFB)_2_-C**^2^H**O (*t*_R_, 10.58 min) was performed. Insert shows the structures of the derivatives. The GC-MS apparatus model TSQ 7000 was used. Redrawn from Tsikas et al. 2016 [18].

The *hd*IE_C_ of the PFB derivatives of malondialdehyde (MDA) (OHC-CH_2_-CHO) and [1,3-^2^H_2_]malondialdehyde (O^2^HC-CH_2_-C^2^HO), i.e., OHC-C(PFP)_2_-CHO and O^2^HC-C(PFB)_2_-C^2^HO is very close to 1.0000 despite the involvement of two D atoms. This is likely to be due to the sterical effect of the two voluminous PFB residues, which avoid almost completely the interaction of the D atoms with the stationary phase of the GC column (Figure 11). This observation suggests that the melting and boiling points may have a lower impact on *hd*IE_C_. 

### 4.5. Possible Implications of Chromatographic Isotope Effects in Metabolomics

GC-MS and GC-MS/MS are mature technologies and widely used for the quantitative determination of known endogenous substances in biological samples. Nowadays, quantitative GC-MS-based analyses are increasingly called targeted-based metabolomics when several analytes are simultaneously analyzed, as described in the present work for amino acids and fatty acids. Targeted GC-MS analyses require the use of isotopologs as an internal standard for “absolute” quantification and commonly one (e.g., fatty acids), two (e.g., amino acids), and even three different derivatization reactions (e.g., prostaglandins). GC-MS is increasingly used in untargeted metabolomics studies, similar to untargeted LC-MS/MS, aiming to discover novel compounds. Analytical protocols combining targeted and untargeted GC-MS metabolomics have been proposed [41]. 

In particular, untargeted metabolomics faces formidable analytical challenges, mainly due to the chemical diversity of physiological substances and their expanding concentration range over several orders of magnitude in biological samples (e.g., pM to mM, i.e., 10^−12^ to 10^−3^ M). Quantitative GC-MS or targeted GC-MS metabolomics referring to a class of analytes, such as fatty acids or amino acids, are based on the use of analytical methods that yield relatively homogenous extracts. Untargeted GC-MS metabolomics are constrained to make analytical compromises at the cost of analytical quality in order to include as many analytes as possible. One potential drawback is the infamous matrix effect, which is especially strong in LC-MS, notably in the *shotgun* mode, i.e., without chromatography. In quantitative GC-MS (i.e., targeted GC-MS metabolomics), the use of isotopologs and specific analytical procedures allows for highly reliable quantitative analyses. Moreover, the chromatographic isotope effect, which is easily available, may be suitable for implementation in quality control (QC) systems as shown for amino acids in human plasma, serum, and urine samples from clinical studies [1]. Consideration of the chromatographic isotope effect will increase the quality assurance as it combines two main closely interrelated quality criteria, which are the retention times of the analytes themselves and their isotopologs. 

For example, study human plasma samples (*n* = 297–353) and QC samples (*n* = 54–64) were analyzed by targeted GC-MS for amino acids within eight runs [42]. *hd*IE_C_ and δ_(H/D)_ values were close in the study and the QC plasma samples and ranged between 1.002 and 1.006, and 0.84 s and 2.64 s, respectively.

## 5. Conclusions

The greatest IE_C_ effects in isotopologs are exerted by the deuterium atoms of the lipophilic analytes, because they interact directly with the mostly hydrophobic stationary phase of fused-silica capillary GC columns. This interaction is weaker than that of the H atoms of the analytes, thus resulting in a weaker retention and shorter retention times of ^2^H-isotopologs. Even a small number of D atoms causes clearly and highly reproducibly quantifiable *hd*E_C_ effects. Even thoroughly ^13^C-labeled analytes do not exert measurable IE_C_ effects, because their “inner” C atoms do not interact directly with the stationary phase of the GC column and their possibly indirect action is rather negligible. This is likely to be true for ^15^N- and ^18^O-isotopologs even for derivatives such as PFB-*NO_2_, PFB-O*NO_2_, and FA-C*O_2_-PFB.

The *hd*IE_C_ effects are clearly observable in derivatives of *cis*- and *trans*-fatty acids and are accompanied by stronger but equally directed *ct*E_C_ effects caused by the *cis/trans*-isomerism thus resembling the *hd*IE_C_ effects. The comparably shorter retention times of the FA-PFB derivatives are the result of an interaction of a structural part of the elongate *trans*-isomers, which is of a shorter duration than that of the curvy *cis*-isomers. Such an explanation is applicable to the epimer stereo-isomerism of prostaglandins. A further explanation for the longer retention times of *cis*-fatty acids could be that the –C=C-double bond is exposed for a longer time to the stationary phase within GC columns. Yet, it must be noted that the *hd*IE_C_ effects are appreciably small with respect to the GC-MS retention times, for instance, about 0.8% for d_6_-metformin (Figure 2).

Protiated and perfluorinated organic solvents and derivatization reagents used in GC-MS analysis such as PFB-Br differ in their boiling points. These differences are transmitted to the derivatized analytes. The H/F effect is stronger than the H/D effect due to the stronger shielding of the analytes by F atoms from the stationary phase, a kind of “Teflon effect”. This is supported by findings by Wang and colleagues who found that benzyl, mono-fluorobenzyl, and di-fluorobenzyl derivatives have longer retention times in GC-ECD than PFB derivatives of 4-hydroxy-acetophenone (E_H/F_ 1.0417, 1.0484, 1.0886, respectively) and an up to seven-fold less strong electron-capture response compared to PFB derivatives due to the higher number of F atoms [43].

Of the so-far-tested stationary GC columns, the ionic-liquid stationary phase IL111i is the only stationary phase that interacts with analytes mainly by π–π, dipole–dipole, and dipole–induced dipole interactions and may be the reason for the elution of deuterated analytes later than their protiated isotopologs [31].

The present work focused on GC-MS, but the chromatographic isotope effects [44] and other types of chromatographic effects such as isomerism [37,45], also apply to LC-MS even to a greater extent [3]. A method has been proposed in HILIC-LC-MS/MS metabolomics for the analysis of fatty acids derivatized with unlabeled and tetradeuterated 2-dimethylaminoethylamine and the authors of that work expected that the proposed approach will improve metabolite annotation in HILIC-MS-based metabolomics analysis [46]. Given the complexity of the chromatography processes and of the biological samples, the plethora of greatly heterogeneous endogenous and exogenous analytes, targeted (i.e., quantitative) GC-MS- and LC-MS-based metabolomics using stable isotope labeled analytes are better prepared for analytical challenges.

The particular analytical challenge for both, GC-MS and LC-MS, namely the analysis of L-amino acids (major fraction), their counterparts D-amino acids (minor fraction), and chiral secondary amino acids, has not been addressed in the present work, not because they are less important from a physiological point of view. Analysis of enantiomeric amino acids and secondary amino acids requires special analytical techniques, notably the use of special chiral columns and derivatization reagents, which are usually performed by targeted, i.e., quantitative GC-MS- and LC-MS-based metabolomics [47,48,49,50]. A potential limitation of the present work is the inclusion of a relatively small number of papers published by authors from other groups because of the lack of retention times of analytes such as polyamines [51].

Deuterium-labeled physiological substances, such as vitamins and polyunsaturated fatty acids, and non-physiological compounds, such as drugs, have been repeatedly used in in vitro and in vivo investigations [51,52,53,54]. For instance, deuterated alpha-tocopherols have been used to study by GC-MS the biokinetics and bioavailability of vitamin E and the role of the liver in their secretion in the plasma [52]. In animal studies, deuterated polyunsaturated fatty acids have been shown to exert protective effects against atherosclerosis by lowering lipid peroxidation and hypercholesterolemia [53], and to reduce hippocampal amyloid β-peptide levels [54]. The underlying mechanisms of the biological activity of deuterium-labeled substances remain elusive. GC-MS and other MS-based methods such as H/D exchange mass spectrometry [55,56] and non-MS techniques, notably NMR [57], should be useful to elucidate such “biological” H/D isotope effects.

## Figures and Tables

**Table 1:** Number of publications in PubMed (https://pubmed.ncbi.nlm.nih.gov) obtained by using the indicated GC-MS and LC-MS search terms alone and in combination (+) with other research terms. Retrieved on 11 December 2024.

Search Term	Articles	Search Term	Articles
GC-MS	103,389	LC-MS	114,147
GC-MS quantitative	11,640	LC-MS quantitative	19,612
GC-MS metabolomics	6923	LC-MS metabolomics	11,553
+targeted	1401	+targeted	3245
+untargeted	902	+untargeted	2335
+nontargeted	442	+nontargeted	833
+quantitative	582	+quantitative	1331

**Table 2:** GC-NICI-MS retention times (*t*_R_) of unlabeled (d_0_Me) amino acids (AAs) as methyl ester pentafluoropropionyl (AA-Me-PFP) derivatives, and isotope effect (*hd*IE_C_) values of unlabeled and deuterium labeled (d_3_Me) amino acid derivatives as measured in urine samples of 29 healthy humans. Numbers in parentheses are the coefficients of variation values. GC-NICI-MS in the SIM mode as described previously [26]. MW, molecular weight.

Amino Acid	MW Amino Acid	MW AA-Me-PFP	*t*_R_ (min)	*hd*IE_C_ Value
Alanine	89	249	3.382 (0.51)	1.005 (0.15)
Threonine	119	425	3.799 (0.23)	1.004 (0.15)
Glycine	75	235	3.794 (0.33)	1.005 (0.07)
Valine	117	277	4.017 (0.22)	1.005 (0.13)
Serine	105	411	4.139 (0.16)	1.006 (0.15)
Sarcosine	89	249	4.491 (0.17)	1.005 (0.08)
Leucine/Isoleucine	131	291	4.644 (0.14)	1.004 (0.07)
Guanidino acetate	117	423	6.258 (0.09)	1.004 (0.08)
Aspartate/Asparagine	133	307	6.200 (0.05)	1.006 (0.04)
Hydroxy proline	131	437	6.400 (0.03)	1.003 (0.03)
Proline	115	275	6.602 (0.06)	1.003 (0.05)
Glutamate/Glutamine	147	321	7.383 (0.06)	1.005 (0.05)
Methionine	149	309	7.393 (0.06)	1.006 (0.06)
Ornithine/Citrulline	132	438	8.099 (0.07)	1.002 (0.00)
Phenylalanine	165	325	8.154 (0.06)	1.002 (0.00)
Tyrosine	181	487	8.578 (0.06)	1.002 (0.04)
Lysine	146	452	9.002 (0.07)	1.002 (0.05)
Arginine	174	626	9.230 (0.05)	1.002 (0.04)
Homoarginine	188	640	10.03 (0.20)	1.003 (0.06)
Tryptophan	204	510	10.92 (0.04)	1.002 (0.04)
Asymmetric dimethylarginine	202	654	11.08 (0.06)	1.002 (0.03)

**Table 3:** Chromatographic isotope effect (*hd*IE_C_) values observed in the GC-NICI-MS analysis of perfluorinated derivatives of biogenic amines, polyamines, other physiological substances and drugs. MO, methoxime; PFP, pentafluoropropionyl; PFB, pentafluorobenzyl; PFBz, pentafluorobenzoyl; TMS, trimethylsilyl.

Analyte	Derivative	*hd*IE_C_ Value	References
d_0_Creatinine	d_0_Crea-(PFP)_1_	1.0010	[7]
d_3_Creatinine	d_3_Crea-(PFP)_1_		
d_0_Histamine	d_0_HA-(PFP)_2_	1.0018	[8]
d_4_Histamine	d_6_HA-(PFP)_2_		
d_0_Metformin	d_0_Metf-(PFP)_3_	1.0082	[9]
d_6_Metformin	d_6_Metf-(PFP)_3_		
Spermidine	SPD-(PFP)_3_	1.0009	[10]
^13^C_4_Spermidine	^13^C_4_SPD-(PFP)_3_		
d_o_Acetylsalicylic acid	d_o_ASA-PFB	1.0028	[11]
d_3_Acetylsalicylic acid	d_3_ASA-PFB		
d_o_Acetazolamide	d_o_AZM-(PFB)_2_	1.0023	[12]
d_3_Acetazolamide	d_3_AZM-(PFB)_2_		
d_o_Dihydroxyphenylglycol	d_o_DHPG-PFB-(TMS)_3_	1.0000	[13]
d_3_Dihydroxyphenylglycol	d_3_DHPG-PFB-(TMS)_3_		
d_o_Dimethylamine	d_o_DMA-PFBz	1.0066	[14]
d_6_Dimethylamine	d_6_DMA-PFBz		
d_o_Epoxyoctadecanoic acid	d_o_EODA-PFB	1.0004	[15]
d_2_Epoxyoctadecanoic acid	d_2_EODA-PFB		
d_o_Ibuprofen	d_o_Ibu-PFB	1.0012	[16]
d_3_Ibuprofen	d_3_Ibu-PFB		
d_0_Hydroxyeicosanoic acid (LTE_4_)	d_0_HEA-PFB-TMS	1.0083 1.0125 1.0021	[17]
d_3_Hydroxyeicosanoic acid	d_3_HEA-PFB-TMS		
d_6_Hydroxyeicosanoic acid	d_6_HEA-PFB-TMS		[17]
^18^O_2_Hydroxyeicosanoic acid	^18^O_2_HEA-PFB-TMS		
d_o_Malondialdehyde	d_o_MDA-(PFB)_2_	0.9999	[18]
[1,3]d_2_Malondialdehyde	d_2_MDA-(PFB)_2_		
d_o_Methylmalonic acid	d_o_MMA-(PFB)_3_	1.0014	[19]
d_3_Methylmalonicic acid	d_3_MMA-(PFB)_3_		
d_o_Paracetamol	d_o_APAP-PFB	1.0008	[20]
d_4_Paracetamol	d_4_APAP-PFB		
d_0_Hydroxynonenal	d_0_HNE-OPFBz-TMS	1.0017	[21]
d_3_Hydroxynonenal	d_3_HNE-OPFBz-TMS		
d_0_Malondialdehyde	d_0_MDA-(OPFBz)_2_	1.0005	[21]
[1,3]d_2_Malondialdehyde	d_2_MDA-(OPFBz)_2_		
d_0_Ethanol	d_0_ETO-(OPFBz)_1_	1.0095	[22]
d_5_Ethanol	d_5_ETO-(OPFBz)_1_		
d_0_Prostaglanin E_2_	d_0_PGE_2-_PFB-MO-(TMS)_2_	1.0010	[23]
[3,4]d_4_Prostaglandin E_2_	d_4_PGE_2_-PFB-MO-(TMS)_2_		
d_0_Arachidonic acid	d_0_AA-PFB	1.0020	[24]
d_8_Arachidonic acid	d_8_AA-PFB		
d_0_Symmetric dimethylarginine	d_0_SDMA-(PFP)_4_	1.0069	[25]
d_6_Symmetric dimethylarginine	d_6_SDMA-(PFP)_4_		

**Table 4:** Retention times (*t*_R_) of unlabeled saturated and unsaturated fatty acid methyl esters (d_0_Me) and of deuterium-labeled saturated and unsaturated fatty acid methyl esters (d_3_Me) and their chromatographic isotope effect (*hd*IE_C_). c, *cis*; t, *trans*. Constructed with data reported by Tintrop et al. 2023 [6].

Fatty Acid	*t*_R_ d_0_Me	*t*_R_ d_3_Me	*hd*IE_C_
C6:0	7.47	7.43	1.0053
C7:0	9.82	9.76	1.0061
C8:0	11.94	11.91	1.0025
C9:0	13.87	13.84	1.0021
C10:0	15.70	15.66	1.0025
C11:0	17.35	17.32	1.0017
C12:0	18.91	18.88	1.0015
C13:0	20.39	20.36	1.0014
C14:0	21.79	21.77	1.0009
C15:0	23.13	23.10	1.0012
C16:0	24.42	24.39	1.0012
C16:0, d31	24.01	-	1.0171
C17:0	25.65	25.63	1.0008
C17:0, d33	25.33	-	1.0126
C18:0	26.82	26.80	1.0007
C20:0	29.05	29.03	1.0007
C21:0	30.12	30.09	1.0010
C22:0	31.14	31.12	1.0006
C16:1c	24.90	24.88	1.0008
C18:1t	27.05	27.02	1.0011
C18:1c	27.16	27.13	1.0011
C18:2c	27.87	27.85	1.0007
C18:3c6	28.39	28.37	1.0007
C18:3c9	28.76	28.74	1.0007
C20:5c	31.61	31.59	1.0006

**Table 5:** Retention times (*t*_R_) of the FA-PFB derivatives of unlabeled and deuterium-labeled fatty acids and their newly calculated *hd*IE_C_ values. The Table was constructed with data reported by Quehenberger et al. 2011 [4]. C, number of carbon atoms; D, number of double bonds. See also Figure 6.

Fatty Acid	C:D	*t*_R_ (min)	*hd*IE_C_
Lauric acid	12:0	3.75	1.0054
	12:0-d_3_	3.73	
Myristic acid	14:0	5.28	1.0019
	14:0-d_3_	5.27	
Pentadecanoic acid	15:0	6.04	1.0016
	15:0-d_3_	6.03	
Palmitic acid	16:0	6.80	1.0029
	16:0-d_3_	6.78	
Margaric acid	17:0	7.54	1.0013
	17:0-d_3_	7.53	
Stearic acid	18:0	8.25	1.0012
	18:0-d_3_	8.24	
Oleic acid	18:1 (ω-9)	8.02	1.0025
	18:1-d_2_	8.00	
Arachidic acid	20:0	9.63	1.0021
	20:0-d_3_	9.61	
Arachidonic acid	20:4 (ω-6)	8.95	1.0022
	20:4-d_8_	8.93	
Eicosapentaenoic acid	20:5 (ω-3)	8.97	1.0022
	20:5-d_5_	8.95	
Behenic acid	22:0	10.92	1.0018
	22:0-d_3_	10.90	
Docosahexaenoic acid	22:6 (ω-3)	10.16	1.0010
	22:6-d_5_	10.15	
Lignoceric acid	24:0	12.14	1.0008
	24:0-d_4_	12.13	
Cerotic acid	26:0	12.91	1.0008
	26:0-d_4_	12.90	

**Table 6:** Fatty acids, *m/z* values of the ions [M-PFB]^−^ used in SIM in GC-NICI-MS analyses, retention times of the FA-PFB derivatives obtained for the indicated *trans*-fatty acids (*t*) and *cis*-fatty acids (*c*), and their ^13^C isotopologs, and calculated *ct*E_C_ values. The Table was constructed with data reported by Kuiper et al. 2018 [3].

Fatty Acid	Trivial Name	*m/z*	*t*_R_ (min)	*ct*E_C_
C16:1n-7 t	Palmitelaidic acid	253.2	77.2	1.0233
C16:1n-7	Palmitoleic acid	253.2	79.0	
^13^C_5_-C16:1n-7 t		258.4	77.2	
C18:1n-9 t	Elaidic acid	281.3	93.7	1.0171
C18:1n-9	Oleic acid	281.3	95.3	
^13^C_5_-C18:1n-9 t		286.4	93.7	
C18:1n-7 t	*trans*-Vaccenic acid	281.3	94.4	
^13^C_5_-C18:1n-7 t		286.4	94.4	
C18:2n-6 t,9 t	Linoelaidic acid	279.3	98.1	1.0163
C18:2n-6,9	Linoleic acid	279.3	99.7	
^13^C_5_-C18:2n-6 t,9 t		284.4	98.1	

**Table 7:** Fatty acids, *m/z* values of the ions [M-PFB]^−^ used in SIM in the GC-NICI-MS analyses, retention times of the FA-PFB derivatives obtained for the indicated regular fatty acids including the *cis*-fatty acids, and their ^2^H and ^13^C isotopologs, and calculated *hd*IE_C_ values. The Table was constructed with data reported by Kuiper et al. 2018 [3].

Fatty Acid	Trivial Name	*m/z*	*t*_R_ (min)	*hd*IE_C_
C14:0	Myristic acid	227.2	58.5	1.0428
D_27_-C14:0		254.4	56.1	
C14:1n-5	Myristoleic acid	225.2	63.9	
C16:0	Palmitic acid	255.3	74.3	1.0000
^13^C_16_-C16:0		271.3	74.3	
C16:1n-7	Palmitoleic acid	253.2	79.0	1.0000
^13^C_16_-C16:1n-7		269.3	78.9	
C18:0	Stearic acid	283.3	91.2	1.0423
D_35_-C18:0		318.5	87.5	
^13^C_18_-C18:1n-9		299.3	95.2	
C18:1n-7	*cis*-Vaccenic acid	281.3	96.4	1.0010
^13^C_5_-C18:1n-7		286.4	96.3	
C18:2n-6,9	Linoleic acid	279.3	99.7	1.0000
^13^C_18_-C18:2n-6,9		297.3	99.7	
C18:3n-6,9,12	γ-Linolenic acid	277.1	100.9	
C20:0	Arachidic acid	311.3	101.6	1.0130
D_39_-C20:0		350.7	100.3	
C18:3n-3,6,9	α-Linolenic acid	277.1	102.2	1.0049
D_14_-C18:3n-3,6,9		291.5	101.7	
C20:1n-9	Gondoic acid	309.3	102.9	
C20:2n-6,9		307.3	105.1	
C20:3n-6,9,12	Dihomo-γ-linolenic acid	305.3	106.6	
C22:0	Behenic acid	339.4	106.7	1.0133
D_43_-C22:0		382.9	105.3	
C20:4n-6,9,12,15	Arachidonic acid	303.3	107.2	1.0009
D_8_-C20:4n-6,9,12,15		311.3	107.1	
C20:5n-3,6,9,12,15	EPA	301.1	109.8	1.0018
D_5_-C20:5n-3,6,9,12,15		306.3	109.6	
C24:0	Lignoceric acid	367.4	111.5	1.0146
D_47_-C24:0		414.9	109.9	
C22:4n-6,9,12,15	Adrenic acid	331.3	113.1	
C24:1n-9	Nervonic acid	365.4	113.2	
C22:5n-6,9,12,15,18		329.3	113.6	
C22:5n-3,6,9,12,15	DPA	329.3	115.9	
C22:6n-3,6,9,12,15,18	DHA	327.3	116.5	1.0017
D_5_-C22:6n-3,6,9,12,15,18		332.3	116.3	

## Data Availability

The study did not report any data.
